# Associated factors of acute primary angle closure glaucoma in a sub-group of Chinese people: comparison between attack eyes and normal controls

**DOI:** 10.1038/s41598-017-14685-2

**Published:** 2017-11-02

**Authors:** Lifang Liu, Xinyu Liu, Chukai Huang, Geng Wang, Di Ma, Wanqi Zhang, Ce Zheng, Mingzhi Zhang

**Affiliations:** Joint Shantou International Eye Center of Shantou University and the Chinese University of Hong Kong, Shantou, China

## Abstract

Acute primary angle closure glaucoma (APACG) is an ophthalmic emergency that may lead to irreversible blindness. Although efforts were made to control intraocular pressure, disease progression still existed. Anterior segment optical coherence tomography (AS OCT) may provide a new insight into mechanism of APACG. In order to seek out associated factors by AS OCT, We compared anterior segment parameters between 74 APACG and 48 normal eyes. Analysis of variance, principle component analysis and logistic regression were used. APACG group had more women (75.7% vs 47.9%, p = 0.002), smaller anterior chamber (anterior chamber depth, ACD; anterior chamber area, ACA; all p = 0.001), narrower angle (AOD500, AOD750, angle opening distance at 500um and 750um; TISA500, TISA750, trabecular-iris space area at 500um and 750um; ARA500, ARA750, angle recess area at 500um and 750um; all p < 0.001), thinner iris (iris thickness at 750um, IT750; maximum of iris thickness, ITMAX; all p < 0.001), larger iris area (IA, p < 0.001) and lens vault (LV, p = 0.005). Principle component extracted were AOD500, AOD750, TISA500, TISA750, ARA500, ARA750, IA, PD (pupillary diameter), ACD, ACA and IT750. After adjusting for confounding factors, AOD750 (b = 12.40 ± 2.56, p < 0.001), IT750 (b = 10.50 ± 3.45, p = 0.002) and IA (b = −1.56 ± 0.77, p = 0.044) were significantly associated with occurrence of APACG.

## Introduction

APACG is characterized by a suddenly increased intraocular pressure (IOP) caused by acute angle closure, If not treated urgently and effectively, it may lead to permanent vision loss. Asian people, female sex, narrow periphery angle, thick lens, anterior lens location and short axial length were known risk factors for APACG^1^. However, even with treatments to eliminate pupillary block, to widen angle or to reduce angle synechia, there remained a significant number of patients requiring medical treatment or secondary operation^2^.

A previous study in White Caucasian individuals found that in a period of 27 ± 14 months, 15% of APACG eyes developed to chronic or late stages, 6% were blinded, and 10% were visually impairment^3^. Another study of APACG in Hong Kong Chinese indicated that at 4 years after attack, 50% had abnormal IOP, 31% had non-improved vision and 11% were blinded^4^. It is imperative to take a new insight into mechanism of APACG, and advance of AS OCT in recent years provides an opportunity to expand our knowledge about APACG.

Moghimi *et al*.^5^ reported that 37.5% of acute angle closure eyes had pupillary block, 50% of them had exaggerated LV, in comparison with fellow eyes and primary angle closure suspect, APACG eyes had the shallowest ACD, least iris curvature and greatest LV. Guzman *et al*.^6^ noted that LV, TISA750 and IT750 were major determinants of APACG. Other studies showed that AS OCT parameters changed differently in normal and angle closure eyes: angle became wider after pharmacological mydriasis in normal individuals^7^, less loss of IA with pupil dilation was verified in APACG, angle closure and primary angle suspect^8–10^, lens vault loss and anterior chamber dimension loss remained smaller in angle closure eyes^8^.

Although several anatomical and dynamic factors of APACG were newly identified by AS OCT, exact parameters that lead to APACG were still uncertain. Mid-dilated pupil was one of the most important feature of APACG^11^, and narrower angle width was the only parameter identified for a significant IOP increase after pupil dilation^12^. This was different from normal individuals, in which angle width widened and 68.9% of IOP decreased in pupil dilated situation^7,13^. Discrepancy between APACG and normal individual in dilated condition hinted that structural or re-distributional difference of anterior segment may exist. While most studies about APACG used fellow eyes of APACG, primary angle closure suspect or primary angle closure as controls, advantages of using normal individuals were as follows: (1) comparison between angle closure and normal open angles may hint new information about angle closure mechanism^8,14,15^; (2) normal eyes can provide a normal reference to both APACG and fellow eyes and screened important parameters associated with APACG^16–18^; (3) fellow eye or primary angle suspect had a risk of inducing high intro-ocular pressure in pupil dilated condition, the risk in normal people was relative low, so normal controls were more frequently used in dynamics of anterior segment^19,20^. Therefore, the purpose of this study was to compare AS OCT parameters between APACG and normal dilated eyes and identify associated factors of APACG in Chinese people.

## Methods

**Figure 1:**
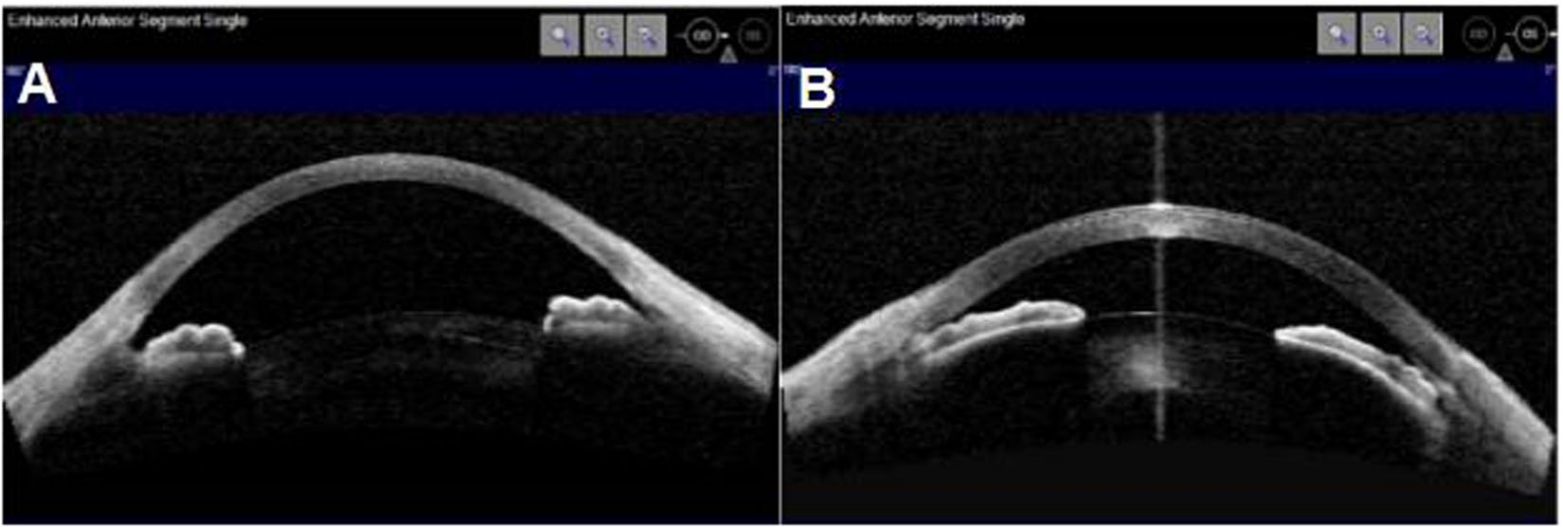
ASOCT images in normal and APACG eye: (**A**) Normal eye (after dilation), pupil diameter = 6.35 mm, ACD = 2.67 mm, LV = 0.36 mm (**B**) APACG eye (after anti-glaucoma drugs), pupil diameter = 3.92 mm, ACD = 1.45 mm, LV = 0.91 mm.

This prospective study was approved by the Ethnics Committee of Joint Shantou International Eye Center of Shantou University and the Chinese University of Hong Kong. The study was conducted in accordance with the principles of the Declaration of Helsinki and the Good Clinical Practice Guidelines. We included 74 APACG eyes and 48 normal eyes between July 2015 and March 2016 at the Joint Shantou International Eye Center of Shantou University and the Chinese University of Hong Kong. Informed consent was obtained from all participants. APACG was defined as^21^: (1) at least two of the symptoms-ocular pain or headache, nausea and/or vomiting; (2) IOP at least 30 mmHg; (3) at least three of the following signs: Conjunctival injection, corneal epithelial edema, fixed mild-dilated pupil, closed/narrow angle, and shallow anterior chamber. Inclusion of normal controls were based on: (1) no history of glaucoma or ocular surgery that may influence anterior segment construction; (2) IOP < 21 mmHg without medication; (3) open angle on gonioscopy; (4) absence of glaucomatous optic nerve damage or visual field damage. After attack, all APACG eyes accepted anti-glaucoma drugs, which included beta-blockers, alpha-2 angonists, topical/systemic carbonic anhydrase inhibitors and miotics. If IOP was higher than 40 mmHg, 250 ml of minnitol was intravenously given. ASOCT images were captured thereafter. To mimic dilated pupil in APACG, pupils were dilated by 0.5% tropic-amide in normal controls before ASOCT images were captured (Fig. 1). Uncooperative individuals or individuals with unclear corneal were excluded. Data of age, sex, laterality and AS OCT measurements were collected in both groups.

All ASOCT (Visante; Carl Zeiss Meditec, Dublin, CA) examinations were done by a single operator, masked to the results of clinical findings. Scans were taken along the horizontal axis using the enhanced anterior segment single protocol in a room without windows, with door closed and the only lighting with the AS OCT screen (<1 lux)^5,22^. After capturing several images, the one with the best quality was chosen for analysis. Images were processed using customized software, the Anterior Segment Analysis Program (ASAP; National University Hospital, Singapore)^8^ by a single experienced observer who was masked to clinical data. The only observer input was to determine the location of the scleral spurs. The algorithm then automatically calculated the anterior segment parameters^23^: AOD500, AOD750, TISA500, TISA750, ARA500, ARA750, IT500, IT 750, ITMAX, IA, PD, ACD, ACW, ACA and LV.

ANOVA and Student’s t-test were used for analysis of continuous variables. Chi-square testing was used for analysis of categorical variables. Principle component analysis was applied to mitigate multicollinearity among anterior segment parameters and identify primary predictors^24^. Binary logistic regression with forward method was used to detect the associated factors for APACG. The level of significance was defined as p-value less than 0.05. All the statistical analyses were performed with using SPSS software version 19.0 (SPSS Inc, Chicago, IL).

## Results

The characteristics of patients in two groups were listed in Table 1. Mean age in APACG (n = 74) and normal group (n = 48) was not statistically different (64.15 ± 7.23 years vs 66.77 ± 11.92 years, p = 0.175). There were more women in APACG group (75.7% vs 47.9%, p = 0.002). Laterality between the two groups was not statistically significant (p = 0.098).

**Table 1:** Comparison of characteristics between APACG patients and normal controls.

	APACG	Normal	p value
N	74	48	
Age	64.15 ± 7.23	66.77 ± 11.92	0.175^#^
Female	56 (75.7%)	23 (47.9%)	0.002^+^
Male	18 (24.3%)	25 (52.1%)	
Left eye	30 (40.5%)	27 (56.2%)	0.098^+^
Right eye	44 (59.5%)	21 (43.8%)	

^#^Independent Sample t-test. ^+^Chi-square test.

Table 2 revealed that nasal and temporal parameters were not statistically different in both normal and APACG group (all p > 0.05). We used temporal data in both groups as representatives in logistic regression. Except for ACW and IT500, all other AS OCT parameters were statistically different between normal and APACG group (all p < 0.05) (Table 3).

**Table 2:** Comparison of nasal and temporal AS OCT parameters in both normal and APACG patients.

Parameters	Normal			APACG		
	Nasal	Temporal	p	Nasal	Temporal	p
AOD500 (mm)	0.16 ± 0.15	0.17 ± 0.16	1	0.007 ± 0.032	0.027 ± 0.072	1
AOD750 (mm)	0.30 ± 0.17	0.33 ± 0.18	1	0.036 ± 0.078	0.059 ± 0.105	1
TISA500 (mm^2^)	0.04 ± 0.05	0.04 ± 0.04	1	0.001 ± 0.007	0.009 ± 0.024	0.88
TISA750 (mm^2^)	0.11 ± 0.09	0.11 ± 0.08	1	0.007 ± 0.020	0.025 ± 0.049	0.482
ARA500 (mm^2^)	0.04 ± 0.05	0.03 ± 0.04	0.807	0.001 ± 0.007	0.008 ± 0.021	1
ARA750 (mm^2^)	0.10 ± 0.08	0.10 ± 0.07	1	0.008 ± 0.020	0.022 ± 0.046	0.603
IT500 (mm)	0.43 ± 0.16	0.38 ± 0.11	0.167	0.31 ± 0.11	0.34 ± 0.11	0.835
IT750 (mm)	0.45 ± 0.13	0.44 ± 0.11	1	0.32 ± 0.10	0.35 ± 0.10	0.761
IA (mm^2^)	1.35 ± 0.25	1.35 ± 0.28	1	1.49 ± 0.34	1.62 ± 0.42	0.136
ITMAX (mm)	0.53 ± 0.10	0.52 ± 0.09	1	0.45 ± 0.09	0.43 ± 0.09	1

Paired sample t-test.

**Table 3:** Comparison of AS OCT parameters between normal and APACG patients.

Parameters	Normal	APACG	P value
AOD500 (mm)	0.17 ± 0.16	0.02 ± 0.06	<0.001
AOD750 (mm)	0.31 ± 0.17	0.05 ± 0.09	<0.001
TISA500 (mm^2^)	0.04 ± 0.05	0.005 ± 0.018	<0.001
TISA750 (mm^2^)	0.11 ± 0.08	0.016 ± 0.038	<0.001
ARA500 (mm^2^)	0.03 ± 0.04	0.004 ± 0.016	<0.001
ARA750 (mm^2^)	0.10 ± 0.07	0.015 ± 0.036	<0.001
IT500 (mm)	0.40 ± 0.14	0.33 ± 0.11	0.092
IT750 (mm)	0.44 ± 0.12	0.34 ± 0.10	<0.001
ITMAX (mm)	0.53 ± 0.10	0.44 ± 0.09	<0.001
IA (mm^2^)	1.35 ± 0.26	1.55 ± 0.38	<0.001
PD (mm)	5.89 ± 1.08	3.96 ± 1.44	0.028
LV (mm)	0.24 ± 0.55	1.17 ± 0.29	0.005
ACW (mm)	11.92 ± 0.45	11.54 ± 0.48	0.24
ACD (mm)	2.66 ± 0.49	1.58 ± 0.25	0.001
ACA (mm^2^)	21.14 ± 4.50	10.42 ± 2.16	0.001

Independent Sample t-test.

Linear regression indicated significant multicollinearity among AS OCT parameters, which was judged by the threshold of VIF^25^ (variance of inflation factors) ≧ 10 (Table 4). Principle component analysis with varimax rotation was used to eliminate multicollinearity and extract predominant variables. Principle component (PC) with eigenvalue ≧ 1 was considered as statistically significant^26^. The first four principle components accounted for 79.24% of total variance. PC1 accounted for 48.28% of total variance, and loaded heavily on angle parameters (AOD500, AOD750, TISA500, TISA750, ARA500, ARA750). PC2 accounted for 14.47% of total variance, which indicated the importance of iris area and pupil diameter. PC3 loaded heavily on anterior chamber parameters (ACD, ACW, ACA), explaining 8.90% of total variance. PC4 was contributed by iris thickness (IT500, IT750), and accounted for 7.58% of total variance (Tables 5 and 6).

**Table 4:** Analysis of multicollinearity among AS OCT parameters.

Predictors	Regression coefficient	Standard error	Variance inflation factors (VIF)
AOD500	−0.13	−1.23	5.64
AOD750	−0.09	−0.93	5.50
TISA500	−6.18	1.43	5.82
TISA750	−0.14	−0.94	11.74*
ARA500	5.24	1.06	10.45*
ARA750	−0.14	−0.89	12.36*
IT500	0.05	1.02	1.06
IT750	−0.09	1.89	1.21
ITMAX	0.68	0.23	1.19
IA	0.01	0.10	1.26
LV	−0.03	−0.37	3.61
ACD	−0.20	−0.93	24.94*
ACW	−0.02	−0.38	1.38
ACA	0.04	0.01	3.54

*VIF > 10.

**Table 5:** Extracted principle component from AS OCT parameters and their account for total variance.

Principle component	Eigenvalue	% of variance	% of cumulative variance
1	7.725	48.28	48.28
2	2.316	14.474	62.754
3	1.425	8.904	71.657
4	1.213	7.58	79.237

**Table 6:** Rotated principle component loading of AS OCT parameters.

Predictors	PC1	PC2	PC3	PC4
SEX	−0.049	0.084	−0.617	−0.244
AOD500	0.918*	0.083	0.182	0.073
AOD750	0.779*	0.319	0.4	0.085
TISA500	0.953*	0.014	−0.03	0.039
TISA750	0.956*	0.148	0.195	0.043
ARA500	0.9*	0.28	0.243	0.047
ARA750	0.965*	0.117	0.154	0.056
IT500	0.022	−0.012	−0.022	0.847*
IT750	0.05	0.224	0.062	0.835*
ITMAX	0.088	0.124	0.419	0.521
IA	−0.08	−0.907*	0.176	0.025
PD	0.166	0.815*	0.172	0.288
LV	−0.352	−0.559	−0.497	−0.197
ACW	0.185	0.017	0.655*	−0.13
ACD	0.466	0.516	0.634*	0.101
ACA	0.477	0.575	0.62*	0.11

Primary predictor variables.

ACW and IT500 were not statistically different between APACG and normal group, so we included Sex, AOD500, AOD750, TISA500, TISA750/ARA500/ARA750/ACD (one at a time to avoid collinearity), IA, PD, ACA and IT750 in Logistic regression analysis, results revealed that after adjusting for sex, AOD500, TISA500, TISA750, ARA500, ARA750, PD, ACD and ACA, AOD750, IT750 and IA were significantly associated with occurrence of APACG (Table 7).

**Table 7:** Logistic regression analysis of determinants of APACG.

Predictors	Regression coefficient (b ± SE)	Variance inflation factors (VIF)	P value
SEX			0.958
AOD500			0.42
AOD750	12.40 ± 2.26	1.129	<0.001*
TISA500			0.533
TISA750			0.766
ARA500			0.283
ARA750			0.629
IA	−1.56 ± 0.77	1.098	0.044*
PD			0.212
ACD			0.443
ACA			0.264
IT750	10.50 ± 3.45	1.063	0.002*

*p<0.05.

## Discussion

Known risk factors of APACG has discovered by A-ultrasound, gonioscopy and ultrasound bio-microscopy for a long time. Shallow anterior chamber, narrow peripheral angle, thick lens, anterior lens position and short axial length were popular reported ones^1^. Although traditional tools helped us to understand basic pathogenesis of APACG, limitations of contact, dis-automated, subjective, time-consuming, un-quantitative were obvious. AS OCT became popular recently for its advantage of sitting positioned, non-contact, fast, semi-automated, objective, high-resolutional, quantitative, high repeatability and reproducibility^27^.

In this study, we found that all AS OCT parameters in APACG group were smaller than normal group except for IA and LV. This was similar to results reported by others. Seager *et al*.^8^ confirmed that IA was significantly larger in angle closure group compared with normal and open angle glaucoma group, and less loss of IA on dilation may contribute to AAC attack. Wang *et al*.^28^ reported that larger IA was significantly associated with narrow angles. Previous studies suggested that greater LV was the prominent feature of APAC affected eyes compared with fellow eyes^29^, primary angle closure suspect^5^, primary angel closure or primary angle closure glaucoma^6^. We guess that zonular laxity, forward movement of lens and iris pushed by posterior chamber pressure, choroidal volume expansion may be the reason for greater LV. Larger LV led to smaller ACD, IA may had an effect on narrower angle, and both of which explained a smaller ACA. This was partly confirmed by our study, data showed that in our study, ACD was negatively correlated with LV (R^2^ = −0.828, P < 0.001), IA was negatively correlated with AOD750 (R^2^ = −0.284, P = 0.002) and TISA750 (R^2^ = −0.18, P = 0.047), correlation between IA and other AS OCT parameters was not statistically significant (all P > 0.05). Furthermore, there was another interesting find-out, IA was negatively correlated with PD (R^2^ = −0.286, P = 0.001). So we speculated that larger IA in APACG led to a narrower angle. According to report by Nongpiur^30^, IA in our study was only medium, this means that smaller or larger IA was relative to its contrast, and a threshold associated with occurrence of APAC may exist. This assumption needs confirmation by others in the future.

However, in Logistic regression analysis, LV was not an independent risk factor for APACG. The first parameter included in forward regression was AOD750. This was also confirmed by other reports. Narayanaswamy *et al*.^31^ assessed the diagnostic performance of angle parameters in 1465 participants, they confirmed that AOD750 was the most significant angle measurement for identifying narrow angles in gradable AS-OCT images: areas under the receiver operating characteristic curves were highest for AOD750 in the nasal (0.90, 95% confidence interval, 0.89–0.92) and temporal (0.90, 95% confidence interval, 0.89–0.92). Nongpiur *et al*.^32^ identified the association between baseline ASOCT parameters and the development of gonioscopic angle closure, findings suggested that smaller AOD750 and larger LV explained 38% of gonioscopic angle closure, for every 0.1 mm decrease in AOD750, the odds ratio of developing gonioscopic angle closure was 3.27 (95% confidence interval, 1.87–5.69). Besides this, several studies have used AOD750 as an index to assess treatment outcomes of APACG^33,34^. The importance of AOD750 in assessment of angle width was also verified by our study: AOD750 was positively correlated with most of angle parameters (AOD500, R^2^ = 0.784, P < 0.001; TISA500, R^2^ = 0.674, P < 0.001; TISA750, R^2^ = 0.903, P < 0.001; ARA500, R^2^ = 0.616, P < 0.001; ARA750, R^2^ = 0.865, P < 0.001; ACW, R^2^ = 0.282, P = 0.002; ACD, R^2^ = 0.474, P < 0.001; ACA, R^2^ = 0.509, P < 0.001), and negatively correlated with LV (R^2^ = −0.356, P < 0.001) and IA (R^2^ = −0.284, P = 0.002).

The second parameter in logistic regression was IT750. Iris and its dynamics was a newly reported parameter in recent studies. Lee and his colleagues observed narrow angles in different ethnics, results hinted that IT750 was both a significant predictor of AOD750 in Whites and Africans and a predictor of TISA750 in Whites^22^. Atalay reported that IT750 was predictive of AOD750 in both APACG affected and fellow eyes^35^. Effect of IT750 on anterior segment was not clear, it made sense that mild-dilated pupil in APACG attack led to thicker peripheral IT and thus narrower angle. This was proved by previous studies. Guzman identified risk factors for APACG, comparison showed that thicker IT750 was a major determinant of APACG^6^. Other reports also pointed out that thinner IT750 was associated with greater increase in angle width after laser peripheral iridotomy^36^. These all indicated that thicker IT750 was detrimental to APACG. However, it cannot be verified by our study. IT750 in our study was thinner than normal controls and previous reported APAC eyes. Although IT750 was positively correlated with AOD750 (R^2^ = 0.224, p = 0.013), the correlation between IT750 and other AS OCT parameters was not statistically significant (all p > 0.05).

The third parameter in logistic regression was IA. It was not frequently reported in studies of APACG. Sng and colleagues described ASOCT parameters in APACG and fellow eyes, they found that IA was smaller in APACG eyes, but it was not an dependent risk factor associated with APAC attack^37^. Nevertheless, IA was verified as a factor associated with occludable angle in Chinese^38^, it was one of six variables that explained variability of TISA750 and AOD750^39^, and it was negatively correlated with change of AOD750 after laser peripheral iridotomy^40^.

We seeked out three associated factors for APACG by ASOCT, but there were still limitations in this study. Firstly, the sample size was not big enough. For logistic regression, the more the variables, the more the sample size. In our study, the variables were 16, so sample size would at least be 80 in both APACG and normal group. Secondly, gender was not balanced between APACG and control group. Although the ratio of female in APACG group (75.4%) was similar to other studies (Male:female = 1:3.3, female ratio, 76%)^3,4^, the female:male ratio in control group was smaller (about 1:1), and data in our study showed that some of angle parameters was statistically different between men and women (ARA500, P = 0.035, IT500, P = 0.044 in APACG group; AOD500, P = 0.043, AOD750, P = 0.039 in control group), this may induce bias, because AOD750 entered into logistic regression. Thirdly, ASOCT images were captured after treatment, so it would not be the real situation of APACG, especially if mitotic drug was used that can affect angle parameters. Nevertheless, APACG was an ophthalmic emergency, images before treatment were not practical and unethical. Pretreatment anterior segment imaging during acute primary angle closure was only reported by two studies^34,37^. AOD750 was smaller than ours (0.002 and 0.002 mm VS 0.05 ± 0.09 mm), IT750 (0.34 and 0.33 mm VS 0.34 ± 0.10 mm) and IA (1.55 and 1.43 mm^2^ VS 1.55 ± 0.38 mm^2^) was similar to ours. Difference may due to sample diversity or drug-induced bias. According to results reported by Sng^37^, before and after medical treatment (same protocol to ours), difference in AOD750, IT750 and IA was 0.02 mm (−0.03 to 0.06), −0.01 mm (−0.05 to 0.02) and 0.27 mm^2^ (0.18 to 0.37) respectively. Therefore, bias induced by anti-glaucoma drugs was 3.03% (0.01/0.33) for IT750 and 18.89% (0.27/1.43) for IA in their study. The bias for AOD750 can not be calculated (0.02/0). Besides, not all patients subsided after treatment, so images may differ between severe and subside patients, further sub-groups should be divided. Fourthly, Pupil were dilated in normal group, the anterior segment especially the angle parameters may not represent the normal controls. The potential bias has been observed by previous studies. Zhang *et al*.^41^ analyzed anterior segment changes induced by tropic-amide in normal Chinese, outcome indicated that AOD500 changed from baseline 0.322 mm to 0.386 mm (19.88% of baseline), IA changed from 2.70 mm^2^ to 1.77 mm^2^ (−34.4% of baseline). Guo *et al* ^42^ compared anterior segment changes before and after mydriasis in normal people, results showed that ∆AOD500, ∆IT750, ∆IA was −0.074 ± 0.111 mm (P = 0.009), −0.09 ± 0.034 mm (P < 0.001), and 0.523 ± 0.162 mm (P < 0.001) respectively. An interesting point was that anterior chamber became wider after mydriasis in normal subjects^43^, which was very different from APACG eyes. This may indirectly verified that iris dynamics in open and angle eyes was different in conditions of pupil dilation, and loss of iris area was smaller in angle closure eyes: loss of IA per mm of PD increase was 0.145 mm^2^ in primary angle closure glaucoma, 0.161 mm^2^ in fellow eyes and 0.165 mm^2^ in normal controls^44^. According to previous results, the corrected IA of normal group in our study should be 1.67  mm^2^ (1.35 + 0.165*(5.89–3.96)) if we take pupil diameter into account. So the difference in pupil diameter between two groups may induce bias. Although pupil diameter has an effect on other AS OCT parameters: in our study, PD was positively correlated with angle width (AOD750, R^2^ = 0.271, P = 0.003; TISA750, R^2^ = 0.195, P = 0.031; ARA750, R^2^ = 0.182, P = 0.045; ACW, R^2^ = 0.246, P = 0.006; ACD, R^2^ = 0.46, P < 0.001; ACA, R^2^ = 0.553, P < 0.001), and negatively correlated with LV and IA (LV, R^2^ = −0.395, P < 0.001; IA, R^2^ = −0.286, P = 0.001), it did not enter into model in Logistic regression. Lastly, all images were captured at one time-point, dynamic changes of anterior segment were not observed. All of these deficiency should be overcame in further studies.

In conclusion, in this study, APACG eyes had smaller anterior anterior chamber and narrower angle but greater IA and LV than normal controls. Greater LV had an effect on smaller ACD, but effect of IA on angle parameter was controversial. AOD750, IT750 and IA were independent associated factors for APACG. Effect of AOD750 was easily understood and confirmed by other studies, IT750 was positively correlated with AOD750, and less loss of IA in pupil dilated situation may be detrimental to angle closure. Further studies with precise design and detailed accomplishment will be needed in the future.
